# Toxicological and environmental evaluation of the two-phase immersion cooling fluid HFO-153-10mczz-E

**DOI:** 10.3389/ftox.2026.1840120

**Published:** 2026-08-25

**Authors:** Grayson Abele, Sarah Smallets, My Hua, Aryatara Shakya, David Brew, Patricia Underwood, Dennis Paustenbach

**Affiliations:** 1 TRC Strategic Health Sciences, Denver, CO, United States; 2 TRC Strategic Health Sciences, Glendale, CA, United States; 3 TRC Strategic Health Sciences, Jackson, WY, United States; 4 TRC Strategic Health Sciences, Washington, DC, United States

**Keywords:** 2-phase immersion cooling, data centers, dielectric fluid, *In vitro* toxicity analysis, *in vivo* toxicity analysis, PFAS (per- and polyfluoroalkyl substances), risk assessment

## Abstract

HFO-153-10mczz-E (decafluoro-3-hexene) is a volatile, nonflammable hydrofluoroolefin (HFO) developed as a dielectric fluid in heat transfer applications, such as two-phase immersion cooling (2-PIC). Its high vapor pressure and low water solubility indicate that inhalation is the primary route of exposure and that if released into the environment, the molecule would primarily partition into the air. The available toxicology dataset primarily comprises studies performed with Good Laboratory Practice (GLP) and under Organisation for Economic Co-operation and Development (OECD) test frameworks. This dataset includes acute, subacute, and subchronic inhalation studies, and reveals low systemic toxicity in rats at concentrations of up to approximately 3,000 ppm, with no treatment-related findings in reproductive or developmental endpoints. An *in vitro* and *in vivo* genotoxicity battery yielded no indication of genotoxicity. Additional studies illustrated no cardiac sensitization in dogs up to 5,000 ppm and no evidence of ocular/skin irritation or skin sensitization. Environmental testing across algae, invertebrates, and fish revealed no adverse effects in algae or daphnia, with the exception of acute toxicity in fish. However, interpretation was constrained by substantial volatility-related loss of the test material. Environmental partitioning, modeled bioaccumulation factors, and physicochemical properties collectively indicate a low bioaccumulation potential. Collectively, the empirical data characterize HFO-153-10mczz-E as a highly volatile fluorinated fluid with a well-defined toxicological profile, with available data suggesting a low human-health hazard and limited environmental exposure potential under plausible use conditions, though further studies may be warranted.

## 1 Introduction

Data centers are an essential component of modern life. High-performance computing, artificial intelligence (AI), cloud infrastructure, and other data-intensive applications have driven substantial increases in server power density. A generally agreed-upon definition for a data center includes the buildings, facilities, and rooms that contain the data servers, telecommunication equipment, cooling equipment, fire suppression equipment, network infrastructure, advanced security systems, and power generation equipment (Zhang et al., 2018; Zhou et al., 2024).

As data centers consolidate workloads into high-density Graphics Processing Unit (GPU) clusters, traditional air-cooling systems are being recognized as inadequate. These types of systems generally cannot maintain thermal stability above approximately 50 kW per rack, which limits their suitability for next-generation computing environments (Kheirabadi and Groulx, 2016; Haghshenas et al., 2023). A life cycle analysis has found that new technologies, such as direct-to-chip (or “cold plate”) cooling or immersion cooling, may reduce blue water consumption by up to 52% in data centers compared to traditional air cooling technologies (Alissa et al., 2025).

A major challenge for data center operators is high energy consumption, particularly the power required for cooling, which accounts for between 30 percent and 50 percent of total facility energy use (Zhou et al., 2024). Because data centers operate continuously and with high power demands, they contribute appreciably to global electricity consumption and carbon emissions. Historically, assessments estimated that data centers accounted for approximately 1.3 percent of global electricity usage in 2010 (Koomey, 2011), increasing to approximately 1.5 percent by 2024 (Chen, 2025), with projections suggesting that demand may approach 10 percent between 2024 and 2030 (Tao and Gao, 2025). These constraints have accelerated the need for alternative cooling strategies that can support higher thermal loads while reducing the operational and energy burdens of conventional air-cooling systems.

As rack densities exceed the approximately 50 kW limit of air cooling, particularly for high-power GPU clusters, hyperscale operators are increasingly implementing immersion cooling solutions, which enable rack densities up to 250 kW (Kheirabadi and Groulx, 2016). Two general categories exist: single-phase and two-phase immersion cooling (2-PIC) (Haghshenas et al., 2023). In both systems, a dielectric fluid replaces air as the heat transfer medium; however, in the case of 2-PIC, all of the heat is captured by the cooling fluid, which simplifies the structure of the cabinet (Haghshenas et al., 2023; Alissa et al., 2025). This review focuses specifically on 2-PIC.

2-PIC has emerged as an efficient approach for high-density computational systems. In these systems, servers are immersed in a dielectric fluid that undergoes a liquid-to-vapor phase change at the component surface, enabling efficient heat removal and substantially higher rack power densities (Kheirabadi and Groulx, 2016; Opteon, 2026a). This approach reduces dependency on mechanically intensive air-cooling infrastructure and offers advantages in thermal performance, operating stability, and overall energy efficiency. Dielectric fluids used in 2-PIC must be electrically non-conductive, thermally stable, non-flammable, and compatible with materials used in server hardware. They must also possess favorable toxicological and environmental profiles, as operational use involves heating, vapor cycling, and periodic fluid exchange in semi-closed systems where worker interactions can occur.

HFO-153-10mczz-E (also referred to as (E)-1,1,1,2,2,5,5,6,6,6-decafluoro-3-hexene, Opteon™ 2P50, or F22E) is a volatile hydrofluoroolefin (HFO), an unsaturated organic compound comprised of hydrogen, fluorine, and carbon, fluid developed for use in 2-PIC (Opteon, 2026b). Its high vapor pressure and low water solubility indicate that inhalation is the primary plausible route of exposure during routine operations, maintenance, or unplanned releases. The compound partitions predominantly into air, exhibits limited potential for accumulation in aqueous systems, and its log K_ow_ value (3.47) suggests that bioaccumulation above commonly referenced regulatory thresholds (European Chemicals Agency, 2025) is unlikely under typical environmental conditions. These physicochemical properties also influence environmental fate: aquatic exposures are expected to be transient, and dominated by volatilization, rather than persistence.

Fluorinated chemistries are the subject of substantial scientific and regulatory interest. HFO-153-10mczz-E meets the Organisation for Economic Co-operation and Development‘s (OECD’s) structural definition of per- and polyfluoroalkyl substance (PFAS) (Organisation for Economic Co-operation and Development, 2018b; 2021). PFAS are comprised of a significant number of compounds, generally divided into long-chain or short-chain as defined by the number of present carbons covalently bonded to fluorine, with the exact number based on the specific categorization of PFAS (U.S. Environmental Protection Agency, 2021). HFO-153-10mczz-E falls under the category of short-chain PFAS, and has significantly different potential effects in humans and the environment relative to legacy long-chain PFAS, such as PFOA or PFOS.

However, the OECD has also stated that this definition is not intended to function as a regulatory hazard classification (Organisation for Economic Co-operation and Development, 2021). Additionally, the compound does not meet the U.S. Environmental Protection Agency’s (USEPA’s) definition for Persistent, Bioaccumulative, and Toxic compounds (U.S. Environmental Protection Agency, 2019). HFO-153-10mczz-E has physicochemical characteristics (i.e., limited environmental persistence due to the compound’s high volatility, limited partitioning to water due to its high Henry’s Law Constant, and observed atmospheric chemistry) that differ substantially from long-chain PFAS historically associated with longer environmental persistence, bioaccumulation, and health effects reported in epidemiological and toxicological studies, though the strength and interpretation of these findings vary across compounds (Lu et al., 2024; Sulbaek Andersen et al., 2024; Paustenbach et al., 2025). Hydrofluorocarbons (HFCs), including the highly prevalent HFC-134a, which have historically been used as refrigerant gases, possess a similar toxicological and chemical profile as HFO-153-10mczz-E, with low potential for mammalian toxicity and a highly volatile nature which limits environmental exposure potential; however, HFCs are also notable for their high global warming potential (GWP) when released into the atmosphere, which has resulted in the transition to the use of HFOs as an alternative with relatively lower GWP (National Research Council, 1996; Tsai, 2005; Glüge et al., 2024; Salierno, 2024). Accordingly, hazard potential, pathways of exposure, and resultant risk associated with HFO-153-10mczz-E must be evaluated based on empirical data, rather than on structural analogy to legacy long-chain PFAS.

Although HFO-153-10mczz-E has been extensively evaluated through Good Laboratory Practices (GLP) studies, including acute, subacute, and subchronic inhalation studies, reproductive and developmental toxicity, genotoxicity, *in vitro* skin and eye irritation and sensitization, environmental testing, and physicochemical testing, these results are not consolidated in the publicly accessible scientific literature. Given the increasing interest in immersion cooling technologies and the need for transparent evaluation of the safety profile of dielectric fluids, an integrated review of the available toxicological and environmental data for HFO-153-10mczz-E is warranted, as well as identification of current data gaps.

The objective of this review is to compile, summarize, and evaluate the totality of currently available data regarding the toxicological, environmental, and exposure-relevant properties of HFO-153-10mczz-E. This includes assessment of acute and repeated-dose inhalation studies, reproductive and developmental toxicity studies, new approach methodologies (NAMs) for mutagenicity and sensitization, cardiac sensitization testing, and aquatic toxicity studies. This review also considers how the physicochemical characteristics of HFO-153-10mczz-E influence the environmental distribution and exposure pathways for persons who work in data centers. Collectively, these data provide a foundation for assessing the toxicology and subsequent risk of HFO-153-10mczz-E relative to its anticipated use as a dielectric fluid for 2-PIC applications in high-density computational systems.

## 2 Materials and methods

We reviewed a suite of toxicology and environmental studies conducted by, or on behalf of, Chemours. These studies included acute, subacute, and subchronic inhalation toxicity, reproductive and developmental toxicity, *in vitro* and *in vivo* genotoxicity assessments, *in vitro* skin sensitization NAMs and eye irritation assays, cardiac sensitization testing, physicochemical property testing, environmental toxicity, biodegradability studies, and bioaccumulation studies.

Each study was reviewed for relevant information, assessed for quality based on its adherence to GLP and relevant guidelines, and ultimately synthesized with all other available studies to draw overall conclusions on the compound.

A targeted literature search was conducted to identify any additional published data relevant to the health or environmental effects of HFO-153-10mczz-E. Searches were performed in major scientific databases (e.g., PubMed, Web of Science, and Google Scholar) using combinations of the chemical’s identifiers (e.g., HFO-153-10mczz-E, decafluoro-3-hexene, Opteon™ 2P50, and F22E). Searches were conducted for publications available up to March 2026. Only one published paper on HFO-153-10mczz-E was identified during the literature search, Sulbaek Andersen et al. (2024), and all other studies reviewed were provided by Chemours.

A Workplace Environmental Exposure Limit (WEEL) committee report on HFO-153-10mczz-E was also reviewed as part of this assessment (Workplace Environmental Exposure Level et al., 2025). The WEEL report summarizes and interprets available toxicology data and provides an independent occupational exposure limit that served as a comparative reference point; however, it did not influence the interpretation of study outcomes in this review. The WEEL was published in 2025.

## 3 Physical, chemical, and environmental properties of HFO-153-10mczz-E

### 3.1 Physicochemical properties

HFO-153-10mczz-E is a colorless, odorless volatile hydrofluoroolefin fluid developed for use in 2-PIC cooling systems. Chemical identification information about this molecule is available in Table 1. The compound has a melting point of < −54 °C and a boiling point of 48.8 °C, consistent with its use in low-temperature 2-PIC cooling. No flash point was observed across a test range of 0 °C–40 °C, indicating that the substance did not exhibit flammability under the conditions tested. The autoignition temperature was reported as 580 °C.

**TABLE 1:** Chemical identification of HFO-153-10mczz-E.

Property	Value
Chemical Name	(E)-1,1,1,2,2,5,5,6,6,6-decafluoro-3-hexene
CAS Number	1256353-26-0
Molecular Formula	C_6_F_10_H_2_
Molecular Weight	264.06 g/mol
Structural Formula	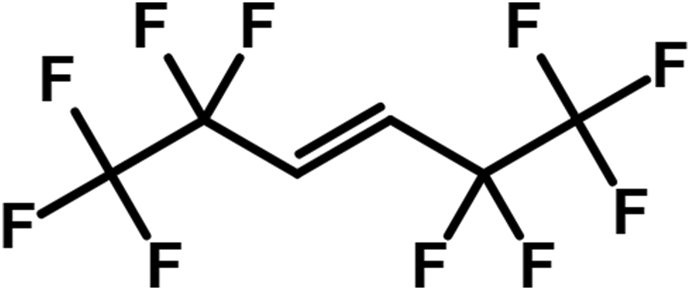
Synonyms	HFO-153-10mczz-E, Opteon™ 2P50, F22E
CAS SciFinder	CAS SciFinder (2024)

Sources: Workplace Environmental Exposure Level et al. (2025), Opteon (2026b).

The maximum water solubility was determined to be 794.1 μg/L. These results confirm that the substance is sparingly soluble in water. The relative density (D420) was measured at 1.463 g/cm^3^, and the surface tension was 69.7 mN/m (ibacon GmBH, 2023).

HFO-153-10mczz-E exhibits high vapor pressure, with reported values of 308 hPa (231 mmHg) at 20 °C, 384 hPa (288 mmHg) at 25 °C, and 1,049 hPa (786 mmHg) at 50 °C. These data indicate that the chemical is highly volatile, with a volatility profile similar to other immersion-cooling fluids, such as 3M’s engineered fluid Novec™ 649, which has a vapor pressure of 402 hPa (303 mmHg) at 25 °C (3M 2022).

HFE-7000, which has a vapor pressure of 537.7 hPa at 20 °C (403 mmHg) (European Chemicals Agency, 2025), also has a comparable recommended 8-hour time-weighted average (TWA) of 250 ppm, compared with HFO-153-10mczz-E’s recommended limit of 200 ppm (European Chemicals Agency, 2025; Workplace Environmental Exposure Level et al., 2025, pg. 128).

Due to this volatility, inhalation is the primary expected route of human exposure, and environmental partitioning is expected to favor air over water or soil.

### 3.2 Partitioning and bioaccumulation potential

The measured log K_ow_ (n-octanol/water) of 3.47 indicates moderate lipophilicity. Although this value has some potential for partitioning into lipid-rich tissues, it is below the commonly used threshold of log K_ow_ = 4.5, above which substances are more likely to exhibit a bioconcentration factor (BCF) ≥ 2,000, the regulatory screening threshold for bioaccumulation concern (European Chemicals Agency, 2008).

Blue Frog Scientific Limited (2025) performed a bioaccumulation assessment of HFO-153-10mczz-E. The authors determined or summarized the partition coefficients of the compound in various media (as summarized below in Table 2) and additionally performed a bioaccumulation factor (BCF) estimation using the Bioaccumulation Assessment Tool (BAT), which accounts for molecular weight, water solubility, Henry’s Law Constant, log K_ow_, log K_oa_, and log K_ow_. The authors estimated that the BCF values for HFO-153-10mczz-E ranged from 234 to 367 L/kg in fish and from 118 to 190 L/kg in invertebrates (Blue Frog Scientific Limited, 2025).

**TABLE 2:** Chemical properties of HFO-153-10mczz-E

Chemical Property		Value
Odor		Odorless
Flash Point		No flash point from 0–40 °C
Auto Ignition Temperature		580 °C
Ready Biodegradability		Day 28 measurement: BOD = 12%, Test Substance = 4%
Water Solubility		794.1 μg/L (at 20 °C)
Partition Coefficients	Octanol/Water (log K_ow_)	3.47
	Octanol/Air (log K_oa_)	1.00 (at 20 °C)
	Cell Membrane Lipid/Water (Aqueous Buffer) (log K_mw_)	Estimated between 2.75 and 3.88 (at 20 °C)
Bioaccumulation Factors	Fish (estimated by BAT)	234–367 L/kg
	Invertebrates (estimated by BAT)	118–190 L/kg

Sources: Consilab Gesellschaft fur Anlagensicherheit mbH (2022a), Consilab Gesellschaft fur Anlagensicherheit mbH (2022e), Consilab Gesellschaft fur Anlagensicherheit mbH (2022b), Consilab Gesellschaft fur Anlagensicherheit mbH (2022f), Consilab Gesellschaft fur Anlagensicherheit mbH (2022d), Consilab Gesellschaft fur Anlagensicherheit mbH (2022c); ibacon GmbH (2022b), ibacon GmbH (2022a), Mitsubishi Chemical Research Corporation (2022), ibacon GmbH (2023), Petro-Lubricant Testing Laboratories Inc. (2023), Blue Frog Scientific Limited (2025).

The authors of the study concluded that, given that the log K_ow_ was less than 4 (measured value of 3.47), the log K_oa_ was less than 5 (measured value of 1), and the BCF predictions from the BAT model were less than 500 L/kg (estimated values between 234 and 367 L/kg), that HFO-153-10mczz-E was not predicted to be bioaccumulative (Blue Frog Scientific Limited, 2025, pg. 24).

Based on the available data, HFO-153-10mczz-E would not be expected to meet the bioaccumulation criteria under typical regulatory frameworks.

### 3.3 Biodegradability

Biodegradability testing (Study No. A220134) was conducted following OECD Test Guideline 301D (Closed Bottle Test) to account for the compound’s volatility (Mitsubishi Chemical Research Corporation, 2022). A decrease in dissolved oxygen due to the decomposition of the test substance by microorganisms in the closed bottles was observed, and the authors calculated the biochemical oxygen demand (BOD) as well as the amount of the test substance remaining. After 28 days, approximately 96 percent of the initial concentration of the test substance still remained, and the average degradability due to BOD was 12 percent. These results indicated that HFO-153-10mczz-E does not meet the OECD criterion for ready biodegradability (≥60 percent degradation based on BOD within 28 days).

However, due to its high volatility, environmental dissipation in real-world scenarios is expected to be governed primarily by air partitioning and physical loss, rather than microbial degradation in aquatic systems.

### 3.4 Environmental fate and atmospheric chemistry

In a test of the compound’s atmospheric chemistry through analysis of airborne reactions with Cl atoms and OH radicals, it was determined that the main atmospheric fate of HFO-153-10mczz-E is a reaction with OH radicals, which resulted in an atmospheric lifetime estimate of 71 and 37 days for the (E)- and (Z)- isomers of the compound, respectively (Sulbaek Andersen et al., 2024). Additionally, the authors determined that the Global Warming Potential (GWP) values of the (E)-isomer were 36, 10, and 3 for time horizons of 20, 100, and 500 years, respectively; the GWP values of the (Z)- isomer were 11, 3, and 1 for the same time frames (Sulbaek Andersen et al., 2024).

## 4 Toxicology of HFO-153-10mczz-E

### 4.1 Acute and subacute toxicity

A series of acute, subacute, and subchronic inhalation studies were conducted to characterize the potential toxicity of HFO-153-10mczz-E in experimental animals. The endpoints evaluated across these studies included mortality, clinical observations, body weight changes, macroscopic pathology, and identification of relevant effect levels (e.g., LC_50_, No Observed Adverse Effect Level [NOAEL], No Observed Adverse Effect Concentration [NOAEC]). No acute toxicity studies were conducted via the dermal or oral routes due to the substance’s physicochemical properties and expected exposure pathways.

#### 4.1.1 Acute dermal toxicity

No acute dermal toxicity study was performed. Given the high volatility of HFO-153-10mczz-E, sustained contact with the skin would not be reliably achieved under testing conditions, and rapid evaporative cooling could induce frostbite-like effects unrelated to its intrinsic chemical toxicity. These factors would confound the interpretation of dermal hazard potential. Skin irritation and sensitization were instead evaluated using validated *in vitro* methods, as described in Section 4.6.

#### 4.1.2 Acute oral toxicity

No acute oral toxicity study was performed. The high volatility of HFO-153-10mczz-E, combined with its intended use in sealed or semi-sealed dielectric cooling systems, indicates that oral exposure is not a credible exposure route under normal or foreseeable occupational conditions. Potential volatilization following ingestion could also produce frostbite-like effects due to evaporative cooling, making conventional oral toxicity testing technically unreliable and difficult to interpret. The available inhalation studies provide the most relevant information regarding systemic toxicity.

#### 4.1.3 Inhalation toxicity

##### 4.1.3.1 Acute inhalation studies

Two acute inhalation studies evaluated the acute toxicity of HFO-153-10mczz-E in rats. In a four-hour whole-body inhalation study, adult male rats (n = 5) were exposed to concentrations of HFO-153-10mczz-E ranging from 6,840 ppm to 12,000 ppm, with a mean concentration of 10,600 ppm (E.I. du Pont de Nemours and Company, 2006). No deaths occurred, and clinical observations during exposure and throughout a 14-day recovery period were unremarkable. Body weight losses of 2.0–7.5 percent were noted 1 day post-exposure, but resolved thereafter, with normal weight gain resuming. Although no testing guideline was reported and no NOAEC was identified, the authors concluded that the approximate lethal concentration exceeded 10,600 ppm (E.I. du Pont de Nemours and Company, 2006, pg. 9).

A more recent guideline-compliant study (OECD Test Guideline [TG] 403; USEPA OPPTS 870.1300) evaluated the acute inhalation toxicity in male and female Sprague Dawley rats exposed to analytically determined concentrations of 1,047, 6,391, 7,664, 9,515, and 21,558 ppm HFO-153-10mczz-E (Charles River Laboratories Ashland LLC, 2022a). No clinical findings or mortality were observed at 1,047 ppm during the exposure or during the 14-day post-exposure observation period. At higher concentrations, rats exhibited concentration-dependent adverse clinical signs, including salivation, reduced respiratory rate, shallow breathing, altered activity, tremors, prostration, convulsions and fur staining (Charles River Laboratories Ashland LLC, 2022a, pg. 25). All animals in the 21,558 ppm group died or were euthanized within 3 minutes of HFO-153-10mczz-E exposure being initiated due to severe clinical signs (Charles River Laboratories Ashland LLC, 2022a, pg. 10, 24).

The NOAEC from this study was identified as 1,047 ppm. Mortality was concentration-dependent, 20 percent at 6,391 ppm, 30 percent at 7,664 ppm, 100 percent in the 9,515 ppm range-finding group, and 100 percent at 21,558 ppm. Using the Litchfield and Wilcoxon method, the 4-hour LC50 was calculated to be 8,074 ppm (range: 6,351–10,264 ppm) (Charles River Laboratories Ashland LLC, 2022a, pg. 24). Based on this value, HFO-153-10mczz-E does not meet the UN Globally Harmonized System (GHS) classification criteria for acute inhalation toxicity for vapors (Category 4 threshold: 20 mg/L (approximately 1,900 ppm equivalent for HFO-153-10mczz-E) (National Research Council, 2014; Organisation for Economic Co-operation and Development, 2018a).

##### 4.1.3.2 Subacute and repeated dose inhalation toxicity

Two repeated-dose inhalation studies evaluated the subacute toxicity of HFO-153-10mczz-E. In a five-day whole-body inhalation study, male and female Sprague Dawley rats were exposed to analytically determined mean concentrations of 313.9 ppm, 1,629.3 ppm, and 3,033.6 ppm HFO-153-10mczz-E for 6 h/day over 5 consecutive days (Charles River Laboratories Ashland LLC, 2022c).

All animals survived until the scheduled necropsy. Across exposure groups, there were no treatment-related clinical signs, changes in food consumption, or clinical pathology findings, and no macroscopic or microscopic abnormalities were observed in the examined organs (Charles River Laboratories Ashland LLC, 2022c). A decrease in mean body weight was observed in male rats across all exposure groups on day 5. The effect was not dose-dependent, with decreases of 4.8 percent in the 1,629.3 ppm group and 3.9 percent in the 3,033.6 ppm group. Comparable decreases were not observed in female rats at the same concentrations (Charles River Laboratories Ashland LLC, 2022c, pg. 23). Based on these findings, the study authors concluded that the no observed adverse effect concentration (NOAEC) for HFO-153-10mczz-E was 3,033.6 ppm.

This study was conducted in parallel with an OECD TG 474 micronucleus assay to evaluate whether the exposure regimen induced micronuclei in polychromatic erythrocytes (PCEs) in rat bone marrow (Charles River Laboratories Ashland LLC, 2022c). The results of that assessment are presented in Section 4.3.

A 28-day repeated-dose study further evaluated inhalation toxicity following exposure to HFO-153-10mczz-E for 6 h/day, 5 days/week for 4 weeks, in accordance with OECD TG 412 (Charles River Laboratories Ashland LLC, 2022d). Achieved mean whole-body exposure concentrations were 301 ppm, 1,501–1,504 ppm, and 2,998–3,003 ppm for males and females, respectively. All animals survived until the scheduled necropsy, and no treatment-related clinical signs were observed. Transient decreases in body-weight gain were reported in high-dose males during early exposure intervals, but these did not persist and were not considered to be biologically meaningful (Charles River Laboratories Ashland LLC, 2022d, pg. 31). Slight increases in mean lung weights and isolated clinical pathology changes lacked clear dose-response relationships and were not supported by histopathology, indicating that they were not toxicologically relevant (Charles River Laboratories Ashland LLC, 2022d, pg. 34). No treatment-related findings were observed in ophthalmologic, neurobehavioral, motor activity, hematology, urinalysis, bronchoalveolar lavage, or gross and microscopic pathology endpoints (Charles River Laboratories Ashland LLC, 2022d, pg. 26, 31–34, 100). The NOAEC for repeated inhalation exposure to HFO-153-10mczz-E was calculated to be 3,003 ppm, which was the highest concentration tested.

##### 4.1.3.3 Subchronic inhalation studies

A 13-week whole-body inhalation study was conducted in Sprague Dawley rats to evaluate the subchronic toxicity of HFO-153-10mczz-E following exposures to analytically determined mean concentrations of 304 ppm, 1,509 ppm, and 3,037 ppm for 6 h/day, 7 days/week, in accordance with OECD TG 413 (Charles River Laboratories Ashland LLC, 2023). The maximum exposure concentration was determined from the NOAEC observed in the aforementioned 28-day repeated-dose study (Charles River Laboratories Ashland LLC, 2022c). Each exposure group consisted of 15 males and 25 females, with a concurrent filtered-air control group. Males were exposed for at least 10 weeks prior to mating and throughout mating (total exposure duration: 90–91 days), while females were exposed for 10 weeks prior to mating and throughout gestation day 20; exposures resumed on lactation day 4, or through post-cohabitation day 24 for unmated females (Charles River Laboratories Ashland LLC, 2023, pg. 12). A reproductive and developmental screening assessment, adapted from OECD TG 421, was conducted concurrently; the results are discussed in Section 4.2 below.

Across all airborne concentrations, no treatment-related mortality or adverse clinical observations were reported. Body weight, food consumption, reproductive performance, thyroid hormone levels, and standard clinical pathology parameters were comparable between the exposed and control groups (Charles River Laboratories Ashland LLC, 2023, pg. 12). Isolated changes were noted, including a minimal increase in urine volume in high-dose females (1.45-fold higher) and higher fluoride levels in high-dose males (1.87-fold higher on days 91/92 and 1.68-fold higher on days 119/120), as well as small reductions in platelet counts in two high-dose animals. These findings were not dose-responsive, lacked microscopic correlates, and were considered by the study authors to be biologically unimportant and unrelated to exposure to HFO-153-10mczz-E (Charles River Laboratories Ashland LLC, 2023).

A statistically significant (p ≤ 0.05) decrease in mean body-weight gain occurred in high-dose males during the post-exposure recovery period (mean gain 25.6 g versus 52.0 g in controls from days 90–118). Because this occurred after exposures had ceased, the effect was not considered to be treatment-related. This effect did not correlate with a significant decrease in food consumption, which suggests the effect may be due to factors other than the treatment. Female body weights and gains during gestation and lactation were similarly unaffected at all exposure levels (Charles River Laboratories Ashland LLC, 2023).

No test-substance-related changes were observed in ophthalmology, neurobehavioral assessments, motor activity, bronchoalveolar lavage, macroscopic pathology, or histopathology (Charles River Laboratories Ashland LLC, 2023, pg. 12). Based on the absence of systemic toxicity at any exposure concentration, the NOAEC for HFO-153-10mczz-E was identified as 3,037 ppm, the highest concentration tested.

A Workplace Environmental Exposure Level (WEEL) Committee later identified this study as the critical basis for derivation of the WEEL value for HFO-153-10mczz-E, establishing a point of departure at 3,000 ppm and applying uncertainty factors to set a recommended occupational exposure limit of 200 ppm (Workplace Environmental Exposure Level et al., 2025, pg. 128).

Collectively, the acute, subacute, and subchronic inhalation studies illustrate that HFO-153-10mczz-E presents a low hazard for short-term and subchronic health effects via inhalation, the primary anticipated exposure route for this highly volatile dielectric fluid. Across study designs ranging from single 4-h acute exposures to 5-day, 28-day, and 13-week repeated dose studies, no mortality or treatment-related systemic effects were observed at concentrations up to approximately 3,000 ppm. This observation appears to be consistent in beagle dogs as well, as documented in Section 4.4. Transient reductions in body weight gain were observed in male rats during the short-term and recovery phases, but these effects were not dose-responsive and were not supported by clinical pathology or histopathology, indicating that they were not toxicologically meaningful. Isolated changes in urine volume, fluoride levels, and platelet counts observed during the 13-week study were similarly sporadic, lacked clear dose-response relationships, and had no corresponding microscopic findings. The consistent absence of treatment-related effects across multiple GLP studies, including a 13-week OECD TG 413 study which identified a NOAEC of 3,037 ppm, the highest concentration tested, provides a coherent line of evidence supporting a conclusion of low inhalation hazard for HFO-153-10mczz-E under plausible exposure conditions.

### 4.2 Reproductive and developmental toxicity

A series of *in vitro* and *in vivo* studies were conducted to assess the potential reproductive and developmental toxicity of HFO-153-10mczz-E. These included a human stem-cell-based developmental toxicity assay, a prenatal developmental toxicity study in rats, and a reproductive/developmental screening study performed as part of a 13-week inhalation study.

#### 4.2.1 *In vitro* developmental toxicity

Stemina Biomarker Discovery (2020) evaluated the developmental toxicity potential of HFO-153-10mczz-E using a human induced pluripotent, not embryonic, stem cell-based assay supported by neonatal human epidermal keratinocytes (HEKn). Cells were exposed for 48 h to airborne concentrations of HFO-153-10mczz-E ranging from 5 ppm to 50,000 ppm (Stemina Biomarker Discovery, 2020, pg. 4). Developmental toxicity potential was assessed using the ornithine-to-cystine (o/c) ratio, a validated metabolic biomarker associated with embryonic development (Stemina Biomarker Discovery, 2019).

Across all exposure concentrations, HFO-153-10mczz-E did not decrease cell viability of iPS or HEKn cells and did not alter the o/c ratio in a manner predictive of developmental toxicity (Stemina Biomarker Discovery, 2020, pg. 1). Although statistically significant variation in normalized viability was observed across concentrations, the magnitude of these differences (approximately 10 percent) was small, not dose-responsive, and not attributed to the test article (Stemina Biomarker Discovery, 2020, pg. 1).

The authors concluded that unmetabolized HFO-153-10mczz-E showed little to no developmental toxicity potential at airborne concentrations up to 50,000 ppm. The WEEL committee independently reached the same conclusion when evaluating this study (Workplace Environmental Exposure Level et al., 2025, pg. 126).

#### 4.2.2 Prenatal developmental toxicity

A prenatal developmental toxicity study was conducted on time-mated Crl:CD (SD) rats exposed to HFO-153-10mczz-E vapor via whole-body inhalation for 6 h/day on gestation days (GD) 6–20, under GLP conditions, following guidelines OPPTS 870.3700 and OECD Guideline 414 (Charles River Laboratories Ashland LLC, 2022e). Analytically verified exposure concentrations were 305 ppm, 1,513 ppm, and 3,015 ppm, with a filtered-air control. Evaluated endpoints included maternal clinical observations, body weights, food consumption, thyroid hormone levels, uterine and thyroid weights, macroscopic and microscopic pathology, intrauterine survival, and fetal morphology (Charles River Laboratories Ashland LLC, 2022e, pg. 11).

All animals survived to the scheduled necropsy, and no test substance-related clinical observations were reported (Charles River Laboratories Ashland LLC, 2022e, pg. 11). Transient reductions in maternal body-weight gain and food consumption were observed in the high-dose group during GD 6–9, but these changes resolved, did not affect overall maternal weight, and were considered non-adverse (Charles River Laboratories Ashland LLC, 2022e, pg. 11).

Fetal endpoints, including post-implantation loss, number of live fetuses, fetal body weights, sex ratios, anogenital distance, and incidence of malformations or variations, showed no evidence of developmental toxicity at any exposure concentration (Charles River Laboratories Ashland LLC, 2022e, pg. 26).

Based on the absence of maternal or developmental toxicity, the NOAEC for both maternal and embryo-fetal toxicity was 3,015 ppm, the highest concentration tested.

#### 4.2.3 Reproductive and developmental screening

A reproductive and developmental toxicity screening study was conducted in conjunction with a 13-week whole-body inhalation study (described above in Section 4.1.3.3) following an OECD TG 421-based study design. As an OECD TG 421 study, this assessment serves as a reproductive and developmental screening evaluation and is not intended to replace a full two-generation reproductive toxicity study. Adult Sprague Dawley rats were exposed to analytically determined concentrations of 304 ppm, 1,509 ppm, and 3,037 ppm HFO-153-10mczz-E for 6 h/day, 7 days/week, prior to mating and through mating, gestation, and early lactation (Charles River Laboratories Ashland LLC, 2023).

In the F0 parental generation, inhalation exposure to HFO-153-10mczz-E at concentrations up to 3,037 ppm did not produce treatment-related effects on survival, clinical observations, body weight, food consumption, clinical pathology, thyroid hormone levels, or reproductive performance, including mating success, pregnancy indices, and gestation length (Charles River Laboratories Ashland LLC, 2023, pg. 12). All evaluated parameters were comparable to controls, and no evidence of systemic or reproductive toxicity was identified in the adult animals.

In the F1 offspring, litter size, viability, and neonatal survival were similarly unaffected by parental exposure. No adverse clinical observations were noted in the pups (Charles River Laboratories Ashland LLC, 2023, pgs. 12–13). Small reductions in mean pup body weights were observed in the 3,037 ppm exposure group, 6.8 percent lower in males on postnatal day (PND) 1 and approximately 5–6 percent lower in male and female pups on PND 13; however, these differences were minimal and were not considered to be adverse by the study authors (Charles River Laboratories Ashland LLC, 2023, pg. 13). Measures of early-life development, including anogenital distance, nipple retention, and thyroid hormone levels, showed no evidence of treatment-related effects.

Overall, the findings indicate that HFO-153-10mczz-E did not produce reproductive or developmental toxicity in either parental animals or their offspring at exposure concentrations up to 3,037 ppm. This exposure level was therefore identified as the NOAEC for systemic, reproductive, and neonatal toxicity (Charles River Laboratories Ashland LLC, 2023, pg. 13).

### 4.3 Genotoxicity and mutagenicity

A series of *in vitro* and *in vivo* studies were conducted to evaluate the potential genotoxicity and mutagenicity of HFO-153-10mczz-E. These included a human TK6 cell-based mode-of-action (MOA) assay, two independent bacterial reverse mutation assays, an *in vitro* chromosome aberration test in human lymphocytes, and an *in vivo* micronucleus assay in rats.

#### 4.3.1 *In vitro* mammalian cell genotoxicity

BioReliance Corporation (2019) evaluated the genotoxic potential of HFO-153-10mczz-E using the Clastogenic, Aneugenic, or Non-genotoxic MultiFlow Assay in TK6 human lymphoblastoid cells. Cells were exposed to a concentration range up to 2,000 μg/mL (10 nM), both with and without S9 metabolic activation (BioReliance Corporation, 2019, pg. 3). Biomarkers of DNA damage, mitotic disruption, and cellular stress (γ-H2AX, phosphor-H3, p53, and nuclear counts) were quantified to predict clastogenic or aneugenic MOAs (BioReliance Corporation, 2019, pg. 3).

Across all concentrations tested, HFO-153-10mczz-E did not induce clastogenic or aneugenic activity, and the assay model predicted a non-genotoxic MOA (BioReliance Corporation, 2019, pg. 3–5). Positive controls performed as expected, confirming assay performance.

#### 4.3.2 Bacterial reverse mutation assays

A bacterial mutagenicity assay was conducted (developed in general accordance with OECD Guideline 471) using multiple *Salmonella typhimurium* strains (TA97a, TA98, TA100, TA1535, and TA1537), and *Escherichia coli* strain WP2 *uvr*A, with and without S9 metabolic activation (BioReliance Corporation, 2021). HFO-153-10mczz-E was tested at eight dose levels up to 1,000 µg/well (BioReliance Corporation, 2021, pg. 13). All strains exhibited normal background lawn growth, and revertant colony counts remained within historical control ranges (BioReliance Corporation, 2021, pg. 4–5). A marginal increase in TA98 was observed, but it was within variability limits and was determined to be not biologically relevant (BioReliance Corporation, 2021, pg. 3). The study concluded that HFO-153-10mczz-E was not mutagenic under the test conditions.

A second GLP-compliant bacterial reverse mutation assay (developed in general accordance with OECD Guideline 471) examined multiple *Salmonella typhimurium* strains (e.g., TA98, TA100, TA1535, and TA1537) and one strain of *E. coli* (WP2 *uvrA*), with and without S9 metabolic activation (Charles River Laboratories, 2022). HFO-153-10mczz-E was tested at six dose levels up to 5,000 μg/plate (Charles River Laboratories, 2022, pg. 13). No treatment-related increases in revertant colonies were observed in any strain, and no cytotoxicity or precipitation occurred (Charles River Laboratories, 2022, pgs. 14–16; 19–20). The study concluded that HFO-153-10mczz-E was not mutagenic in the Ames test (Charles River Laboratories, 2022, pg. 20).

#### 4.3.3 *In vitro* mammalian chromosome aberration test

Charles River Laboratories Den Bosch BV (2024a) evaluated the potential of HFO-153-10mczz-E to induce structural or numerical chromosome aberrations in cultured human lymphocytes following OECD TG 473 and the corresponding Japanese regulatory guideline. Human peripheral blood lymphocytes, stimulated with phytohaemagglutinin, were exposed to HFO-153-10mczz-E for 3–24 h in the absence of S9 metabolic activation and for 3 h in the presence of an S9-mix (Charles River Laboratories Den Bosch BV, 2024a, pg. 8). HFO-153-10mczz-E was tested at six concentrations, ranging from 39 μg/mL to 1,250 μg/mL, the latter representing the limit of solubility (Charles River Laboratories Den Bosch BV, 2024a, pg. 16).

Two cytogenic assays were performed as part of the same study to evaluate the clastogenic potential of HFO-153-10mczz-E at the highest concentrations achievable in culture (i.e., 313 μg/mL, 625 μg/mL, and 1,250 μg/mL). In the first assay, lymphocytes were exposed for 3 h, followed by a 24-h expression period in the absence or presence of 1.8 percent (v/v) S9-mix (Charles River Laboratories Den Bosch BV, 2024a, pgs. 13; 16). In the second assay, designed to extend the exposure duration, cells were treated for 24-h, also followed by a 24-h expression period in the absence or presence of 1.8 percent (v/v) S9-mix (Charles River Laboratories Den Bosch BV, 2024a, pgs. 13; 16). The second cytogenetic assay was performed with a longer exposure time due to clear negative results in the presence of metabolic activation in the first assay (Charles River Laboratories Den Bosch BV, 2024a, pg. 13). All acceptance criteria for the chromosome aberration assay were met, including adequate mitotic indices, acceptable solvent control values, and robust responses in positive control cultures (Charles River Laboratories Den Bosch BV, 2024a, pg. 13). The study was therefore considered valid according to OECD TG 473 requirements.

The report concluded that HFO-153-10mczz-E did not disturb mitotic processes or cell cycle progression, that it did not induce structural or numerical chromosome aberrations, and that it was not clastogenic in human lymphocytes under the experimental conditions (Charles River Laboratories Den Bosch BV, 2024a, pg. 17).

#### 4.3.4 *In vitro* mammalian gene mutation (CHO/HPRT) assay

Inotiv (2025) assessed the potential of HFO-153-10mczz-E to induce forward mutations at the hypoxanthine-guanine phosphoribosyl transferase (HPRT) locus of Chinese hamster ovary (CHO) cells following OECD Guideline 476. Acetone was chosen as the vehicle since the test substance was found to be soluble in it up to 500 mg/mL, as determined in 1.8 mL vials to minimize headspace. A preliminary toxicity test was performed to identify test concentrations. Ten separate concentrations were tested in the preliminary analysis, and it was found that at 2,000 μg/mL, the osmolality of the test substance (292 mmol/kg) was less than 120% of the osmolality of the test vehicle (297 mmol/kg), which was considered acceptable. Additionally, relative survival rates at concentrations from 3.91 to 2,000 μg/mL ranged from 101.52% to 137.36% without S9 activation and from 97.59% to 156.14% μg/mL with S9 activation, respectively (Inotiv, 2025).

Based on the preliminary analysis, the test concentrations were determined to be 125, 250, 500, 1,000, and 2,000 μg/mL. Cells were treated for 5 ± 0.5 h in the presence and absence of S9 in sealed 75-cm^2^ culture flasks. Following the 5-h treatment, the cultures were washed twice with Ca/Mg-free Hank’s Balanced Salt Solution (CMF-HBSS), then trypsinized and counted to determine cell density. Cells were then subcultured in 10 mL of mM L-glutamine and 5% (v/v) heat-inactivated and dialyzed fetal bovine serum. The plates were incubated for 7 days, and the resulting colonies were additionally counted. The cultures were subcultured for 7 days at 2- to 3- day intervals to allow for logarithmic growth and to permit expression of the mutant phenotype.

The average adjusted relative survival rates based on dose (with and without S9 activation, respectively) were 75.80% and 91.22% for 125 μg/mL; 71.20% and 94.23% for 250 μg/mL; 56.74% and 84.70% for 500 μg/mL; 53.85% and 87.15% for 1,000 μg/mL; and 48.47% and 83.19% for 2,000 μg/mL (Inotiv, 2025). No significant increases in mutant frequency were reported relative to the control groups. Based on the conditions of the report, HFO-153-10mczz-E was determined to be negative for the induction of forward mutations at the HPRT locus of CHO cells, both with and without S9 activation.

#### 4.3.5 *In vivo* micronucleus assay

Charles River Laboratories Ashland LLC (2022c) also assessed the potential for HFO-153-10mczz-E to induce micronuclei in PCEs in rat bone marrow following whole-body inhalation exposure for 5 days (previously described in Section 4.1.3.2). Male and female Sprague Dawley rats were evaluated for the percentage of micronucleated PCEs and for bone marrow cytotoxicity (PCE/erythrocyte ratio).

No statistically significant increases in micronucleated PCEs were observed at any exposure concentration, and there was no evidence of bone marrow cytotoxicity (Charles River Laboratories Ashland LLC, 2022c, pg. 24). The study concluded that HFO-153-10mczz-E was negative for clastogenic activity and did not disrupt the mitotic apparatus *in vivo* (Charles River Laboratories Ashland LLC, 2022c, pg. 24).

Across multiple test systems, including human lymphoblastoid cells, human lymphocytes, bacterial mutagenicity assays, and in *vivo* micronucleus test, HFO-153-10mczz-E did not induce gene mutations, chromosomal aberrations, clastogenicity, aneugenicity, or micronucleus formation. These concordant results across GLP and guideline-based assays provide consistent evidence illustrating that HFO-153-10mczz-E does not exhibit genotoxic or mutagenic activity under the tested conditions.

### 4.4 Cardiac sensitization

Cardiac sensitization is a well-recognized hazard associated with several hydrocarbons, including certain fluorinated compounds. This endpoint refers to a transient increase in myocardial susceptibility to catecholamine-induced arrhythmias following chemical exposure. As described by European Centre for Ecotoxicology and Toxicology of Chemicals (2009), sensitization occurs when inhaled chemicals lower the arrhythmogenic threshold such that physiologic or pharmacologic surges of endogenous catecholamines can trigger potentially serious cardiac arrhythmias. Historic reports of sudden deaths following misuse (i.e., “sniffing”) of chlorofluorocarbon aerosol propellants have been reported since the 1960s (Brock, 1998).

Screening for cardiac sensitization potential is conducted using the adrenaline-challenged dog model (ASTM International, 2017). In this model, chemicals are ranked by the lowest inhaled concentration that elicits epinephrine-induced arrhythmias. Brock (1998) reported that CFC-11 sensitized at 0.5 percent (v/v), CFC-12 at 5 percent (v/v), and CFC-115 at 15 percent (v/v), corresponding to “strong”, “moderate”, and “weak” sensitizers, respectively.

Charles River Laboratories Ashland LLC (2022b) conducted a cardiac sensitization study to evaluate whether HFO-153-10mczz-E induced epinephrine-triggered arrhythmias in male beagle dogs. Responses to an epinephrine challenge dose were collected 2 days prior to the study in 10 dogs, and a dose of 8 μg/kg was established for all test animals (the highest challenge dose) as none of the test animals exhibited significant cardiac effects at such exposure. Six dogs were exposed via muzzle-only inhalation to 0.3 percent (3,000 ppm) and subsequently to 0.5 percent (5,000 ppm) HFO-153-10mczz-E for 10 min per exposure, with a 48-h washout interval between exposures. Analytically determined concentrations were 3,050 ppm and 4,809 ppm. Immediately following each exposure, dogs received a bolus intravenous injection of epinephrine (8 μg/kg) to assess arrhythmogenic susceptibility (Charles River Laboratories Ashland LLC, 2022b, pg. 9).

No epinephrine-induced arrhythmias were observed at either test concentration. The authors reported that five dogs exhibited slight tremors and one exhibited intermittent urination at 5,000 ppm; however, no cardiac effects were observed, and no physiological effects were noted at 3,000 ppm (Charles River Laboratories Ashland LLC, 2022b, pg. 9). Due to the absence of sensitization at 0.5 percent (5,000 ppm), higher concentrations were not evaluated. The no-observable-effect-concentration (NOEC) for cardiac sensitization was therefore identified as 0.5 percent (5,000 ppm) (Charles River Laboratories Ashland LLC, 2022b, pg. 9).

The adrenaline-challenged dog model is intentionally conservative. Epinephrine doses used in the assay exceed normal physiological levels and are designed to produce a maximal arrhythmogenic challenge, ensuring that any modest lowering of the sensitization threshold becomes detectable (European Centre for Ecotoxicology and Toxicology of Chemicals, 2009). Within this context, the absence of sensitization at 5,000 ppm provides strong evidence that HFO-153-10mczz-E does not pose a cardiac sensitization hazard under the exposure concentrations tested.

Overall, this study did not identify cardiac sensitization as a target endpoint for HFO-153-10mczz-E. Minor, non-specific clinical signs observed at 5,000 ppm were not associated with arrhythmias and were not indicative of cardiac effects. These findings, combined with the conservativism of the test model, support the conclusion that HFO-153-10mczz-E possesses low potential for epinephrine-induced cardiac sensitization during acute inhalation exposure.

### 4.5 Eye irritation

An *in vitro* eye irritation study was conducted to evaluate whether HFO-153-10mczz-E required classification for eye irritation or serious eye damage under the GHS (Institute for In Vitro Sciences Inc, 2021; United Nations, 2023). The study followed the OECD TG 492 framework, which uses reconstructed human corneal epithelial tissue to assess ocular irritation potential through measurement of tissue viability (Organisation for Economic Co-operation and Development, 2024).

The EpiOcular™ Human Cell Construct (MatTek Corporation) was used to assess the cytotoxicity via the MTT reduction assay following a 30-min exposure to undiluted HFO-153-10mczz-E and a 120 ± 15-min post-exposure incubation. Under OECD TG 492 classification criteria, a relative tissue viability greater than 60 percent indicates that a substance does not require classification as an eye irritant, whereas viability below this threshold triggers assignment to a UN GHS irritancy category (Organisation for Economic Co-operation and Development, 2024).

The study met all OECD TG 492 validity criteria, including an acceptable optical density range for the negative control and a positive control viability of ≤50 percent, and no protocol deviations were noted (Institute for In Vitro Sciences Inc, 2021, pg. 6). The authors reported that a killed-control experiment was not required because HFO-153-10mczz-E did not reduce MTT in the absence of viable tissue, and a colorant-control experiment was not required, as no spectral interference with the MTT assay was anticipated (Institute for In Vitro Sciences Inc, 2021, pg. 7).

The mean relative viability of tissues exposed to HFO-153-10mczz-E was 106.8 percent, exceeding the 60 percent threshold for non-classification, and no ocular irritation or cytotoxicity was observed under the experimental conditions (Institute for In Vitro Sciences Inc, 2021). Based on these findings, HFO-153-10mczz-E was classified as UN GHS No Category for eye irritation, indicating that it does not require hazard classification for ocular irritation under the test conditions.

### 4.6 Skin irritation and sensitization

A series of *in vitro* assays were conducted to evaluate the potential for HFO-153-10mczz-E to cause skin irritation or sensitization, consistent with the OECD test framework for such classifications. These studies included evaluations of skin irritation using a reconstructed human epidermis model (OECD TG 439), assessment of skin sensitization using the Defined Approach (DA) in OECD TG 497, and supporting mechanistic testing covering protein reactivity (DPRA), keratinocyte activation (ARE-Nrf2 luciferase assay), and structure-based predictions.

#### 4.6.1 Skin irritation

Skin irritation, defined under OECD TG 439 as reversible local skin damage following chemical exposure, was assessed using the EpiDerm™ reconstructed human epidermis model (Institute for In Vitro Sciences Inc, 2022b; Organisation for Economic Co-operation and Development, 2025b). Tissues were exposed to 30 µL of undiluted HFO-153-10mczz-E for 60 ± 1 min, followed by a 42 ± 2 h post-exposure incubation (Institute for In Vitro Sciences Inc, 2022b, pg. 7). The assay quantified tissue viability using the MTT reduction method, with 5 percent sodium dodecyl sulfate as the positive control and sterile Ca++/Mg++ Free DPBS as the negative control (Institute for In Vitro Sciences Inc, 2022b, pgs. 8, 11).

Under OECD TG 439, substances are classified as UN GHS No Category for skin irritation if mean tissue viability exceeds 50 percent following exposure (Organisation for Economic Co-operation and Development, 2025b, pgs. 9–10). The mean viability for HFO-153-10mczz-E was 98.15 percent ±3.30 percent, and all assay acceptance criteria were met (Institute for In Vitro Sciences Inc, 2022b, pg. 8). No protocol deviations were reported. Based on these results, HFO-153-10mczz-E was classified as UN GHS No Category for skin irritation.

#### 4.6.2 Skin sensitization

Skin sensitization potential was evaluated using the OECD TG 497 “2-out-of-3” defined approach (DA), which integrates data across at least two key events (KEs) in the skin sensitization adverse outcome pathway: KE1 (protein binding; OECD 442C), KE2 (keratinocyte activation; OECD TG 442D), and KE3 (dendritic cell activation; OECD TG 442E) (Organisation for Economic Co-operation and Development, 2025a, pg. 19). HFO-153-10mczz-E was tested in assays corresponding to KE1 and KE2, and structural analysis was used to complement the DA evaluation.

##### 4.6.2.1 Direct peptide reactivity assay (KE1: protein binding)

Covalent peptide binding was assessed using the DPRA assay (Institute for In Vitro Sciences Inc, 2022a). Two assays were performed. In the first, a cysteine solvent control failed, so only lysine data were considered valid; in the second assay, all measurements met the acceptance criteria. Mean peptide depletion values were 0.46 percent for cysteine and 0.10 percent for lysine, yielding a combined mean depletion of 0.28 percent (Institute for In Vitro Sciences Inc, 2022a). According to the DPRA prediction model, this level of depletion corresponds to minimal reactivity and classifies HFO-153-10mczz-E as a non-sensitizer for KE1 (Institute for In Vitro Sciences Inc, 2022a).

##### 4.6.2.2 ARE-Nrf2 luciferase assay (KE2: Keratinocyte activation)

Keratinocyte activation was assessed using the ARE-Nrf2 luciferase reporter assay (Institute for In Vitro Sciences Inc, 2022a). Three definitive tests were performed, each using a set of four test plates, at 12 total concentrations ranging from 0.977 µM to 2000 µM (Institute for In Vitro Sciences Inc, 2022a, pg. 10). Cinnamic aldehyde served as the positive control. One trial was invalidated due to an inadvertent plate handling error; however, the second and third tests met all acceptance criteria (Institute for In Vitro Sciences Inc, 2022a).

Across valid trials, HFO-153-10mczz-E did not induce luciferase activation consistent with sensitization. EC1.5 values exceeded 2,000 μM, and no cytotoxicity was observed up to the highest tested concentration (IC30 and IC50 > 2,000 µM), and the maximum induction ranged from 1.01 to 1.32, which were well below the threshold indicating pathway activation. The assay, therefore, indicated non-activation of KE2 (Institute for In Vitro Sciences Inc, 2022a, pg. 11). Because KE1 and KE2 assays were both negative, the DA classification was negative without requiring KE3 testing.

##### 4.6.2.3 QSAR structural analysis

QSAR Toolbox analysis (v. 4.5) indicated no structural alerts for protein binding, and skin metabolism/autooxidation models predicted no formation of pre- or pro-haptens for HFO-153-10mczz-E (Institute for In Vitro Sciences Inc, 2022a).

Across all *in vitro* assays and the OECD TG 497 DA, HFO-153-10mczz-E did not exhibit activity consistent with skin sensitization. KE1 and KE2 assays produced concordant negative results, and no structural alerts for sensitization were identified. Based on this integrated evidence, HFO-153-10mczz-E was classified as UN GHS Not Classified for skin sensitization.

### 4.7 Environmental/aquatic toxicity

Because HFO-153-10mczz-E is highly volatile and strongly partitions to air, aquatic exposures under environmental conditions are expected to be transient and short-lived. Nevertheless, three aquatic toxicity studies were conducted under GLP following OECD TGs, using sealed or low-headspace systems to the extent possible. As described in OECD Guidance Document 23, testing of volatile substances presents inherent challenges relating to maintaining exposure concentrations, interpreting effect thresholds, and ensuring exposure integrity (Organisation for Economic Co-operation and Development, 2019). These constraints are relevant to the studies summarized below.

#### 4.7.1 Acute immobilization in *Daphnia magna*

Charles River Laboratories Den Bosch BV (2024c) conducted a 48-h acute immobilization test in *Daphnia magna* following OECD TG 202 and OECD GD 23 under GLP conditions. Twenty daphnids per concentration were exposed in sealed vessels to nominal concentrations of 0.19, 0.41, 0.91, and 2.0 mg/L (Charles River Laboratories Den Bosch BV, 2024c, pg. 13). Mean measured concentrations for the 0.19 mg/L test were 0.15 mg/L at the start of testing and 0.019 mg/L at 48 h, the 0.51 mg/L test concentrations were 0.038 mg/L at the start and 0.10 mg/L at 48 h, the 0.91 mg/L test concentrations were 0.39 mg/L at the start and 0.18 mg/L at 48 h, and the 2.0 mg/L test concentrations were 0.77 mg/L at the start to 0.64 mg/L at 48 h (Charles River Laboratories Den Bosch BV, 2024c, pg. 16). No immobilization occurred in any treatment group, and two solvent-control animals were immobile (<10 percent) (Charles River Laboratories Den Bosch BV, 2024c, pg. 8), which met OECD TG 202 validity criteria (Organisation for Economic Co-operation and Development, 2004). As no treatment-related immobility was observed, the EC50 could not be calculated. The authors reported a 48-h EC50 of >0.70 mg/L, based on geometric mean measured concentrations (Charles River Laboratories Den Bosch BV, 2024c, pg. 19). Because HFO-153-10mczz-E is highly volatile, closed test vessels were required, and higher concentrations were considered impractical to maintain by the study authors (Charles River Laboratories Den Bosch BV, 2024c).

#### 4.7.2 Acute toxicity to *Cyprinus carpio*

Acute fish toxicity was evaluated in a 96-h static-renewal study in *Cyprinus carpio* following OECD TG 203 (Charles River Laboratories Den Bosch BV, 2024d). Seven fish were exposed per concentration to nominal values of 0.1, 0.22, 0.46, 1.0, and 2.2 mg/L in sealed, minimal-headspace vessels with 24-h solution renewal. A range-finding study illustrated increased pigmentation and abnormal swimming behavior at 1.0 and 2.0 mg/L, with one mortality occurring in each of these treatments (Charles River Laboratories Den Bosch BV, 2024d).

Measured concentrations varied substantially over time due to volatility, decreasing within each 24-h interval and across renewals. For example, measured concentrations for the nominal 1.0 mg/L group ranged from 0.36 to 1.2 mg/L. TWA exposures were therefore derived (i.e., 0.070, 0.15, 0.35, 0.75, and 1.1 mg/L) (Charles River Laboratories Den Bosch BV, 2024d).

Concentration-dependent mortality was observed, with no mortality reported at 0.070 mg/L or 0.15 mg/L, 6/7 deaths occurred at 0.35 mg/L, 5/7 deaths occurred at 0.75 mg/L, and 6/7 animals at 1.1 mg/L were euthanized due to severe clinical signs. Abnormal swimming behavior was noted at concentrations >0.35 mg/L, and excess abdominal gas accumulation was noted in a majority of animals in the 1.1 mg/L group by 48 h (Charles River Laboratories Den Bosch BV, 2024d). The authors calculated a 24-h LC50 of 0.29 mg/L (95 percent CI: 0.22–0.39 mg/L) and identical 48-, 72-, and 96-h LC50 values of 0.26 mg/L (95 percent CI: 0.21–0.32 mg/L). Under UN GHS criteria, these values meet the classification threshold for “Acute Category 1: Very toxic to aquatic life” (Charles River Laboratories Den Bosch BV, 2024d).

As noted in the study report and OECD GD 23, the extreme volatility of HFO-153-10mczz-E resulted in substantial declines (>80 percent in many cases) between renewals, contributing to uncertainty regarding true exposure concentrations and complicating interpretation of the quantitative toxicity estimates.

#### 4.7.3 Toxicity to freshwater algae (*Raphidocelis subcapitata*)

Charles River Laboratories Den Bosch BV (2024b) evaluated algal growth inhibition using *Raphidocelis subcapitata* following OECD TG 201 under no-headspace conditions. A combined limit/range-finding test used nominal concentrations of 0.061, 0.20, 0.63, and 2.0 mg/L HFO-153-10mczz-E over 72 h was conducted to quantify the NOEC, EC_10_, EC_20_, and EC_50_ for both growth rate inhibition and yield inhibition. Six replicates were used for the control and limit concentrations. Growth rate inhibition was <4 percent, and yield inhibition was <21 percent across all test groups (Charles River Laboratories Den Bosch BV, 2024b, pg. 18).

A definitive test was subsequently conducted. Measured concentrations decreased rapidly: the 2.0 mg/L limit concentration declined to 6.2 percent of the initial value after 24 h (0.070 mg/L) and to 1.2 percent after 72 h (0.013 mg/L) (Charles River Laboratories Den Bosch BV, 2024b, pg. 18). Based on these measurements, the authors calculated a 72-h TWA exposure concentration of 0.11 mg/L. No statistically significant inhibition of growth rate or yield was observed at this TWA concentration (Charles River Laboratories Den Bosch BV, 2024b, pg. 8). The reported 72-h NOEC was 0.11 mg/L, with EC10, EC20 and EC50 values reported as >0.11 mg/L (Charles River Laboratories Den Bosch BV, 2024b, pg. 8). Interpretation of these findings is limited by the approximately 99 percent loss of HFO-153-10mczz-E under the tested conditions, which is consistent with the volatilization-related challenges described in OECD GD 23 (Organisation for Economic Co-operation and Development, 2019).

Across aquatic species tested under GLP and OECD guidelines, HFO-153-10mczz-E exposure did not cause immobilization in *Daphnia magna* at concentrations up to 2.0 mg/L. Acute toxicity to fish (*Cyprinus carpio*) with LC50 values in the 0.26–0.29 mg/L range (UN GHS Acute 1 classification), and no growth inhibition in freshwater algae at a 72-h TWA of 0.11 mg/L.

In all studies, the compound’s high volatility resulted in rapid and substantial loss of the test material from the test systems, requiring the use of closed systems and leading to high variability in measured concentrations. As noted in OECD GD 23, these conditions introduced methodological uncertainty into the quantitative interpretation of aquatic endpoints for volatile substances (Organisation for Economic Co-operation and Development, 2019). Additionally, closed systems may overestimate potential hazards in non-closed systems, such as environmental release, and may be considered to represent unrealistic conditions. Thus, while exposure likelihood is low, the risk of acute toxicity to fish could potentially be further investigated to assess hazard potential in extreme situations, such as large-scale spills.

## 5 Discussion

HFO-153-10mczz-E is a highly volatile HFO developed for use in 2-PIC systems. Although it meets the OECD structural definition of PFAS, its volatility, low water solubility, and low measured and modeled bioaccumulation potential differ significantly from those of long-chain perfluoroalkyl acids. These characteristics, combined with the available GLP toxicology and environmental health data, provide empirical findings on which the hazard assessment of the compound should be based.

### 5.1 Human health hazard profile

Inhalation is the primary anticipated exposure route for HFO-153-10mczz-E, given its high vapor pressure and intended use. Dermal and oral exposures are not expected to be significant exposure routes, given its anticipated use conditions, and would be difficult to assess meaningfully because of the compound’s properties. Dermal exposure potential is also anticipated to be minimized through the use of personal protective equipment when handling the compound.

Across acute, subacute, and subchronic inhalation studies, systemic toxicity was minimal. No treatment-related findings were identified in clinical observations, clinical pathology, or histopathology at concentrations up to and including approximately 3,000 ppm, and transient body-weight changes found following subchronic exposures lacked a dose-response pattern and toxicological significance. The NOAEC of 3,037 ppm from the 13-week study served as the basis for the WEEL Committee’s recommended 200-ppm exposure limit (Workplace Environmental Exposure Level et al., 2025), further supporting a low inhalation hazard at plausible workplace exposures.

Reproductive and developmental studies conducted under guideline protocols did not identify maternal, fetal, or neonatal toxicity at the highest test concentrations. Minimal pup-weight reductions lacked adversity and dose-response characteristics, and an *in vitro* developmental assay likewise showed no perturbation at concentrations far exceeding plausible human exposures. Overall, the available data for HFO-153-10mczz-E indicate that there are low reproductive and developmental hazards within the tested range.

Genotoxicity testing across bacterial, mammalian cells, and *in vivo* systems consistently produced negative results. The concordance of these findings reduces concern for a genotoxic mode of carcinogenic action, although chronic carcinogenicity studies have not been conducted, and non-genotoxic mechanisms cannot be ruled out. Nevertheless, the evidence suggests that HFO-153-10mczz-E does not exhibit genotoxicity, nor is there evidence to indicate potential carcinogenicity under the tested conditions.

Cardiac sensitization was not observed in the epinephrine-challenge dog model at concentrations of up to 5,000 ppm, a concentration selected based on previously performed studies to avoid potentially confounding central nervous system effects in rats and dogs, despite the assay’s conservative design. *In vitro* evaluations under OECD test frameworks also did not identify eye irritation, skin irritation, or skin-sensitization potential. These results indicate that irritation, sensitization, and cardiac endpoints are not expected to pose a human health hazard for HFO-153-10mczz-E.

Collectively, the human-health evidence for HFO-153-10mczz-E shows a consistent pattern of low hazard across relevant toxicological endpoints, particularly in light of its anticipated use as a cooling fluid. Further data may supplement these conclusions.

### 5.2 Environmental hazard and exposure context

The environmental behavior of HFO-153-10mczz-E is dominated by rapid air-water partitioning. Water solubility is low, and environmental releases are expected to dissipate primarily through volatilization rather than persist in aquatic or soil compartments, which is supported by the substance’s high Henry’s Law Constant.

Aquatic studies identified no immobilization in *Daphnia magna* and no growth inhibition in algae at measured concentrations. Acute fish toxicity produced LC50 values in the 0.26–0.29 mg/L range, meeting UN GHS Acute Category 1: Very toxic to aquatic life criteria. These results describe intrinsic aquatic hazard under laboratory conditions; however, the extreme volatility of HFO-153-10mczz-E resulted in significant concentration loss in sealed and low-headspace systems, consistent with the challenges outlined in OECD Guidance Document 23 (Organisation for Economic Co-operation and Development, 2019).

This volatility drives large uncertainties in quantitative interpretation of the available data. The available data indicates that, if the compound were to be released into the environment at quantities that resulted in dissolution into water despite rapid volatilization, it could be acutely toxic to fish. Such scenarios should inform the way the compound is handled, particularly in terms of transportation and disposal. The data also underscore that long-term environmental exposure scenarios are not plausible for this compound, given that it is far more likely to partition into air than into water where it has been shown to degrade within 71 and 37 days for the (E)- and (Z)- isomers of the compound, respectively (Sulbaek Andersen et al., 2024). Further studies may be warranted to investigate the extent to which, in an unanticipated environmental exposure scenario, HFO-153-10mczz-E may remain in water long enough to pose a hazard to aquatic life.

Modeled BCF factors (supported by experimental partition coefficient results and QSAR predictive modeling) were well below relevant thresholds for bioaccumulation potential, and the measured log K_ow_ of 3.47 does not indicate concern for bioconcentration under typical environmental scenarios. Although the compound is not readily biodegradable under OECD 301D, environmental dissipation is expected to occur primarily through volatilization rather than aquatic degradation pathways.

HFO-153-10mczz-E is anticipated to be used in closed immersion cooling systems with periodic fluid supplementation and industrial handling controls, which limit potential environmental exposure except under potential unintentional release conditions (e.g., accidental spills). Volatility further restricts the duration of any incidental aquatic contact, although localized impacts from accidental releases cannot be fully ruled out in unlikely circumstances given the available acute toxicity data. Thus, intrinsic aquatic toxicity exists for fish, but environmental exposure likelihood is constrained by the compound’s physical behavior and typical use patterns.

### 5.3 Alternatives assessment considerations

Alternatives assessment is increasingly relevant for procurement, sustainability programs, and regulatory screening processes that require demonstration of why a material was selected over other potential options. For 2-PIC systems, functional requirements, including low boiling points (40 °C - 60 °C), high dielectric strength, nonflammability, and material compatibility, limit feasible substitutes to a narrow class of high-volatility dielectric fluids.

Traditional 1-PIC fluids, such as synthetic oils or esters, do not meet the thermophysical requirements for 2-PIC architectures and are therefore not considered viable replacements (Kheirabadi and Groulx, 2016; Roe et al., 2022). The dataset for HFO-153-10mczz-E is robust and aligns with the low-hazard profiles reported for other volatile fluorinated fluids used in electronics thermal management. Thus, based on currently available information, HFO-153-10mczz-E represents a likely suitable dielectric fluid with a well-characterized hazard profile.

### 5.4 Uncertainties and implications for use

Some uncertainties include limited information on human toxicokinetics and endocrine-disruption potential. There are also methodological limitations in the aquatic dataset stemming from volatility-driven concentration instability. There are limited data available on potential occupational exposure concentrations and real-world release estimates. Chronic toxicity data is not available, however the lack of indications of genotoxicity for the compound shows that a chronic carcinogenicity study in animals is not warranted. Additionally, all available toxicity studies were performed on behalf of Chemours, but each has been evaluated by the authors for methodological rigor and adherence to standard protocols.

Within the operational parameters of 2-PIC systems, the integrated evidence, including low inhalation hazard at up to approximately 3,000 ppm, negative reproductive/developmental and genotoxicity findings, lack of cardiac sensitization, low irritation and sensitization potential, limited bioaccumulation potential, and high volatility that restricts environmental exposures, supports the conclusion that HFO-153-10mczz-E poses limited health effects in humans at concentrations at or below 200 ppm. A full risk assessment would need further information regarding potential occupational exposure concentrations.

## 6 Conclusion

The available GLP toxicology, reproductive/developmental, and cardiopulmonary studies consistently indicate that HFO-153-10mczz-E presents a low human health hazard.

### 6.1 Acute and subacute toxicity

The data obtained indicate that the compound is defined as “Practically Non-toxic” Under the Hodge and Sterner Scale for (e.g., LC_50_ between 5,000 and 15,000 ppm). These data were obtained in mice and rats, but appeared to be consistent in beagle dogs as well. The data were only obtained from inhalation studies, but given the properties of the chemical it is unlikely that oral or dermal exposures would occur under anticipated use conditions.

### 6.2 Subchronic toxicity

The data obtained from a 13-week study in Sprague Dawley rats did not result in any observed adverse effects up to 3,037 ppm in air, the highest concentration tested. Body weight, food consumption, reproductive performance, thyroid hormone levels, and standard clinical pathology parameters were all taken into consideration. Some effects on urine parameters and body weight gain were observed, but not determined to be toxicologically relevant to the test article. Taken holistically, the compound has been shown to have low potential for subchronic toxicity and, by inference, chronic toxicity.

### 6.3 Genotoxicity and mutagenicity

Based on the suite of genotoxicity and mutagenicity studies performed on HFO-153-10mczz-E, exposure to the chemical did not induce gene mutations, chromosomal aberrations, clastogenicity, aneugenicity, or micronucleus formations in *vitro* and *in vivo* tests. Given the breadth of cell studies performed, there are no data to suggest that HFO-153-10mczz-E has mutagenic or genotoxic potential in humans.

### 6.4 Chronic toxicity

There were no available chronic toxicity data. Traditional 1- or 2-year animal chronic toxicity or carcinogenicity tests might be informative. However, in the modern era it is generally accepted that chronic toxicity can be predicted via several *in vitro* tests as well as 90 days *in vivo* data supporting the 3 Rs framework to reduce animal testing where possible. If any of those tests are suggestive of biologically important genotoxic effects, then more advanced animal or *in vitro* tests may be warranted. For this chemical, given that there is no information that it is genotoxic or clastogenic, we consider the *in vitro* test data to be an adequate representation of the chronic hazard. Furthermore, under the EPA’s guidance regarding adoption of New Approach Methods (NAMs) for assessing these endpoints rather than traditional vertebrate animal tests; the 2 years chronic bioassay is discouraged. This chemical is not expected to be used outside an enclosed system; therefore human contact would be limited to occasional manipulation of the system.

### 6.5 Reproductive and developmental toxicity

Based on the available *in vitro* and *in vivo* data, exposure to the test compound did not result in any adverse reproductive or developmental effects. The strongest data come from the 13-week study in Sprague Dawley rats where exposures up to 3,037 ppm in air did not result in any adverse effects in either the parent or offspring generations. Based on these studies, the compound is not anticipated to pose a reproductive or developmental hazard to humans.

### 6.6 Cardiac sensitization

Only one study was evaluated regarding cardiac sensitization potential for HFO-153-10mczz-E. The test animals were canines which are known to be more representative of human exposures for this endpoint. Concentrations of HFO-153-10mczz-E up to 5,000 ppm did not result in cardiac sensitization, though there were minor clinical signs that were not indicative of cardiac effects. The available data indicate that HFO-153-10mczz-E poses low potential for epinephrine-induced cardiac sensitization following acute inhalation exposure.

### 6.7 Eye irritation

One *in vitro* eye irritation study was conducted under the OECD TG 492 framework. HFO-153-10mczz-E did not reduce the mean relative viability of tissues below the test threshold, and no ocular irritation or cytotoxicity was observed in the study. Based on the UN GHS classification guidelines, the data indicate that HFO-153-10mczz-E should not be classified as an eye irritant and does not require hazard classification for that endpoint.

### 6.8 Skin irritation and sensitization

The data obtained from the *in vitro* skin irritation and sensitization assays, performed consistent with the relevant OECD test frameworks, thus, HFO-153-10mczz-E was not classified as a skin irritant or sensitizer under UN GHS classifications.

### 6.9 Environmental and aquatic toxicity

Environmental testing indicated low toxicity to daphnids and algae, as well as high acute toxicity to fish, under laboratory conditions. However, the interpretation of aquatic endpoints is constrained by substantial volatility-related loss of test material. The compound’s rapid air–water partitioning, low water solubility, and low model estimated bioaccumulation potential indicate limited environmental persistence under realistic use scenarios. In short, the data suggest that it should pose a low ecological risk under anticipated use conditions.

Given the intended purpose of use, the toxicology of the compound is sufficiently understood for exploration of use as a 2-PIC fluid.

Like other products being brought to market, it is likely that EPA would expect that additional toxicology tests will be conducted to further ensure the lack of public health hazard. This would be consistent with both EPA and European Chemicals Agency (ECHA) guidance provided under the Registration, Evaluation, Authorisation and Restriction of Chemicals (REACH) program.

While some data gaps remain, they are generally associated with limitations of testing the compound due to its physical and chemical properties in combination with the lack of applicable test systems (e.g., the challenges of testing aquatic toxicity of a compound with high volatility and low water partitioning). Similarly, studies on human toxicokinetic parameters were not collected due to the challenges of conducting such studies due to its extreme volatility. This substance, based on its intended use cases, is not anticipated to enter the water supply.

Overall, the current evidence supports the conclusion that HFO-153-10mczz-E has a low hazard profile for human health and a limited likelihood of long-term persistence in environmental compartments due to rapid volatilization and atmospheric degradation.
